# Author Correction: Redundancy and the role of protein copy numbers in the cell polarization machinery of budding yeast

**DOI:** 10.1038/s41467-023-42928-6

**Published:** 2023-11-07

**Authors:** Fridtjof Brauns, Leila Iñigo de la Cruz, Werner K.-G. Daalman, Ilse de Bruin, Jacob Halatek, Liedewij Laan, Erwin Frey

**Affiliations:** 1 Arnold Sommerfeld Center for Theoretical Physics and Center for NanoScience, Department of Physics, Ludwig-Maximilians-Universität München, Munich, Germany; 2 Kavli Institute for Theoretical Physics, University of California Santa Barbara, Santa Barbara, CA 93106 USA; 3 Department of Bionanoscience, Kavli Institute of Nanoscience Delft, Delft University of Technology, Delft, the Netherlands; 4 Max Planck School Matter to Life, Hofgartenstraße 8, D-80539 Munich, Germany

**Keywords:** Biological physics, Nonlinear phenomena, Cell polarity, Evolutionary theory

Correction to: *Nature Communications* 10.1038/s41467-023-42100-0, published online 16 October 2023

In this article the author name Leila Iñigo de la Cruz was incorrectly written as Leila M. Iñigo de la Cruz. The original article has been corrected.

