# Supplementary material for: Redundancy and the role of protein copy numbers in the cell polarization machinery of budding yeast
Source: Nat Commun. 2023 Oct 16;14:6504. doi: 10.1038/s41467-023-42100-0 (PMC10579396; doi:10.1038/s41467-023-42100-0)
Supplement: Supplementary file 1 — Supplementary Information [file 41467_2023_42100_MOESM1_ESM.pdf]

Supplementary Information for

Redundancy and the role of protein copy numbers in the cell polarization machinery of budding yeast

F. Brauns, L. Iñigo de la Cruz, W. Daalman, I. de Bruin, J. Halatek, L. Laan, E. Frey

This PDF file includes

Supplementary Notes 1 to 6

Supplementary Figures S1 to S7

Supplementary Tables S1 to S7

Supplementary References

Other supplementary material for this manuscript includes

Supplementary Movies 1 to 6

Source Data

Contents

1 **Extended model discussion** **3**
- 1.1 Remarks on vesicle-based transport 3
- 1.2 Extensions beyond previous models 4

2 **Linear stability analysis in spherical geometry** **7**
- 2.1 Laterally homogeneous steady states 7
- 2.2 Linearized dynamics 8
- 2.3 Spatial eigenmodes and finding their growth rates 9

3 **Numerical simulations** **11**

4 **Inference of reaction rate parameters** **12**

5 **Supplementary Discussion** **20**
- 5.1 Previous experiments explained by our model 20
- 5.2 Hypothetical feedback via Rsr1 and secondary GEFs 22

6 **Experiments** **22**
- 6.1 Growth frequency assay (Fig. 3) 22
- 6.2 Determination of fitness effects of sfGFP-CDC42 sandwich fusion 23
- 6.3 Size and viability assays under variable expression of CDC42 25
- 6.4 Multinucleated and dead cells 28

1 Extended model discussion

A cursory description of the model is given in the Methods section of the main text. Here we discuss some of the model assumptions in further detail.

1.1 Remarks on vesicle-based transport

Vesicles are transported towards exocytosis sites along polarized actin cables. In budding yeast, the formation of such actin cables is induced by the formin Bni1 which in turn is recruited by Cdc42-GTP. The collective effect of these processes lead to directed transport of vesicle-bound proteins (Cdc42, for instance) to membrane sites of high Cdc42-GTP concentration. Moreover, it was found that Cdc42-GTP activates the exocyst tethering complex and thus promotes vesicle exocytosis in the polar zone independent of actin cables [1]. However, the role of vesicle-based Cdc42 transport for cell polarization remains under debate [2–5].

Vesicle recycling might also indirectly promote Cdc42 polarity. First, it was found that specific lipids (phosphatidylserine) are transported to the polar zone by vesicles and that these lipids in turn promote Cdc42 clustering, activation and membrane-binding [6]. This might result in Cdc42 transport to the polar zone via cytosolic transport and by trapping Cdc42 (see paragraph *Putative recruitment of Cdc42 to the polar zone* below). Second, vesicle recycling has been shown to be important for septin-ring formation by diluting septins in the center of the polar zone [7]. Because septins recruit the GAP Bem2, this effectively transports Bem2 away from the center of the polar zone. Thus, vesicle recycling might have a stabilizing effect on Cdc42 polarity by reducing Cdc42 deactivation within the polar zone [8].

In our reaction–diffusion model, we incorporate directed transport by vesicle recycling as an effective recruitment process of cytosolic Cdc42-GDP to the membrane. This may seem counterintuitive at first. On closer inspection, however, it turns out that both processes share the same key features that are relevant for the Cdc42-polarization machinery. In fact it has been shown experimentally, that the cytosolic transport directed by membrane recruitment and vesicle-based transport directed by formins and the exocyst complex are functionally interchangeable:

- A Bem1-Snc2 fusion chimera, which is permanently membrane bound and transported on vesicles, rescues *bem1*Δ *rsr1*Δ mutants [2, 9]. This shows that diffusive transport of Bem1 to the polar zone due to recruitment by Cdc42 can be replaced by transport on vesicles along directed actin cables (“vesicle recycling”).
- A Cdc24$^{\mathrm{PB}\Delta}$-Snc2 fusion chimera which does not interact with Bem1 and is transported on vesicles completely bypasses the Bem1-mediated recruitment pathway for Cdc24 [10]. In the same study, it is shown that a Cdc24$^{\mathrm{PB}\Delta}$-Cla4 fusion chimera that is directly recruited to Cdc42-GTP bypasses Bem1-mediated recruitment as well. In their summary, the authors explicitly point out the interchangeability of transport pathways: “*Thus, the functional deficit of a Cdc24 that lacks the PB1 domain can be rescued by linkage to a polarized protein, whether that protein polarizes by diffusion capture (Cla4) or vesicle recycling (Snc2).*”

Underlying the functional interchangeability of cytosolic transport and vesicle-based transport in the Cdc42-polarization machinery is that both are directed by Cdc42-GTP, as illustrated in Fig. 1B,C in the main text. On a coarse-grained level, cytoskeletal transport can be described by an advection term which is directed by the local net polarization, $\mathbf{p}$, of cytoskeletal filaments (actin cables)

$$\partial_t^{\text{vesicles}} c = -\nabla \mathbf{J}_{\text{vesicles}}, \quad \mathbf{J}_{\text{vesicles}} \propto c\,\mathbf{p} \tag{1}$$

Importantly, diffusion can also be written in a closely related form, by explicitly writing out the diffusive flux in terms of the concentration gradient

$$\partial_t^{\text{diff}} c = -\nabla \mathbf{J}_{\text{diff}}, \quad \mathbf{J}_{\text{diff}} = D_c \nabla c \tag{2}$$

Thus the gradient replaces the local cytoskeletal polarization. In the cell polarization machinery of budding yeast, the polarization of the cytoskeleton is established and maintained by a localized concentration of Cdc42-GTP (via formins). Similarly, protein recruitment to the membrane, which sets up and sustains cytosolic concentration gradients, is downstream of Cdc42-GTP. Therefore, we can assume $\mathbf{p} \propto \nabla c$, such that cytoskeletal transport effectively is described by a diffusion equation. Hence, in an *effective* description, vesicle-based transport and cytosolic diffusion directed by membrane-recruitment are equivalent and can be modeled in the framework of bulk-surface coupled reaction–diffusion dynamics.

1.2 Extensions beyond previous models

Cdc42-GAP complex. Extending previous models [11, 12], we explicitly incorporate transient formation of a Cdc42-GAP complex as an intermediate step in the enzymatic interaction between GAPs and Cdc42 [13]. This is a crucial difference to previous models that have modeled GAPs in the Ccd42-polarization machinery [7, 14, 15] but have not accounted for the Cdc42-GAP complexes. The finite dissociation rate of the intermediate complex is the reason for enzyme saturation, a generic property of enzymatic kinetics. Saturation is strong if the catalysis and dissociation of the substrate-enzyme (here, Cdc42-GAP) complex is the rate limiting step of the enzyme kinetics, i.e. it is slow compared to the binding step. Indeed, it has been shown that this is the case for GAP-catalyzed hydrolysis of human Cdc42 in budding yeast [13]. One potential experimental test for GAP saturation is to measure the residence time of Cdc42 on the membrane, e.g. via FRAP on the different regions. Slow dissociation of the Cdc42-GAP complexes would imply that the residence time in the polar zone is much longer than elsewhere on the membrane. Unfortunately, it is hard to execute this experiment in a quantitatively convincing manner because of the size of budding yeast. Typical FRAP areas comprise a large part of the yeast cell making it difficult to quantitatively disentangle the polar zone from the rest of the membrane.

In budding yeast, four GAPs for Cdc42 are known: Bem2, Bem3, Rga1, and Rga2 [16]. It has been found that the different GAPs have specific roles in several cellular functions coordinated by Cdc42 polarization, such as pheromone response [17, 18], axial budding [19], and the timing of bud emergence [20]. For the purpose of our model,

we disregard their differences and conflate them into a single effective GAP-species. We account for cycling of GAP proteins between two states: free and Cdc42-bound, both on the membrane. The fact that a fraction of the GAPs is sequestered in these complexes plays a key role in the rescue mechanism. Importantly, in our model, the GAP protein dosage is an explicit parameter and the Bem3 deletion is accounted for by decreasing the dosage of Bem3. Incorporating more states of the GAPs (e.g. a cytosolic state, phosphorylation, etc.) and shuttling of GAPs to internal membranes [18] is not required to explain the rescue pathway for *bem1*Δ cells and goes beyond the scope of the present work.

Finally, let us briefly address the question of the mobility of free GAP proteins. GAP saturation can facilitate Cdc42 polarization as long as resupply of GAPs in the incipient polar zone is not faster than accumulation of Cdc42. This resupply could happen via diffusion of the cytosolic fraction of GAPs. Experiments show that only about 10% of Bem3 is cytosolic and that Bem3 membrane binding is mediated by a PH domain which binds directly to phospholipids in the membrane. While Rga1/2 are not known to have a PH domain, they localize to the bud tip and bud neck during cell polarization and budding [21]. We therefore assume that a significant fraction of them is bound to the membrane (or cortex) during polarity establishment such that a fast resupply of free GAPs in the incipient bud-site which would relieve GAP saturation there is unlikely. Moreover, since GAPs are significantly larger than Cdc42 (ca 10x larger molecular weight), their diffusion will be significantly slower than that of Cdc42. Thus, even if there is a significant pool of cytosolic GAPs, their diffusion would be too slow to relieve GAP saturation. These qualitative statements could be quantified by extending the model to account for GAP attachment and detachment at the membrane. However, this would be a distraction from the core topic of redundant functional submodules. We therefore leave such model extensions for future work and simply assume that the GAPs are membrane bound.

Putative recruitment of Cdc42 to the polar zone. There is some experimental evidence that several downstream effectors of Cdc42-GTP, such as Cla4, Gic1/2, and flippases, may mediate membrane-recruitment of Cdc42-GDP. These recruitment mechanisms are likely to drive Cdc42 transport towards the polar zone independently of vesicle-based transport and independently of Bem1: Cla4 has been found to catalyze the release of Cdc42-GDP from its GDI (Rdi1) [22]. Gic1/2 have been found to stabilize Cdc42-GTP on the membrane, reducing detachment and lateral diffusion away from the polar zone [23–25]. Flippases downstream of Cdc42 flip specific phospholipids to the inner membrane leaflet. These phospholipids have been reported to decrease the detachment of Cdc42 [26]. Moreover, phosphatidylethanolamine, a phospholipid that promotes Cdc42-extraction from the membrane by Rdi1 is flipped to the outer leaflet of the membrane in the polar zone [26]. This effectively reduces Cdc42 detachment in the polar zone compared to the remaining membrane surface.

While the molecular details and functional relationships remain elusive, several experimental findings provide indirect evidence for these putative self-recruitment pathways of Cdc42. First, an overexpression of Gic1 but not of Gic2 was found to partially

rescue *bem1*Δ cells [27]. Conversely, it was recently found that overexpression of Gic2 is lethal for *bem1*Δ *bem3*Δ cells [28]. Second, *gic1*Δ *gic2*Δ *cla4*Δ triple-mutants were reported to grow very slowly [29]. Moreover, this study showed that *gic1*Δ *gic2*Δ mutants are highly temperature sensitive (strongly impaired growth at 35 °C) and that overexpression of Cla4 can suppress the growth defect of *gic1*Δ *gic2*Δ mutants at 35 °C. Finally, also overexpression of Bem1 or of Cdc42 in its WT form, but not in the GTP-locked state (G12V), were found to suppress the growth defect of *gic1*Δ *gic2*Δ mutants at 37 °C [27].

A recent study established that the growth defects in *gic1*Δ *gic2*Δ mutants at high temperature are in fact due to impaired Cdc42-polarization [24]. Strikingly, in this study it was also found that *gic1*Δ *gic2*Δ cells at 37 °C quickly assume a rescue mutation that restores the wild-type behavior. This mutation reproducibly happens at a single locus, its exact identity remains unknown though [24].

Together, these experimental findings suggest that Gic1/2 and Cla4 provide several, potentially redundant pathways of effective Cdc42 self-recruitment feedback loops that become relevant at high temperature to "support" or entirely replace the Bem1-mediated polarization mechanism. In our model, such feedback loops are accounted for by the generic self-recruitment term with rate k_{tD}. Studying the details of these feedback mechanisms remains an open task for experimental and theoretical studies.

GEF membrane binding. Because Cdc24 has its own membrane-binding (PH) domain [30], we also incorporate Cdc24 membrane binding independently of Bem1. Compared to Cdc24 in a complex with Bem1, free membrane-bound Cdc24 has an approximately 50% lower GEF activity towards membrane-bound Cdc42 [31, 32]. We assume that, in the absence of Bem1, Cdc24 will have linear attachment–detachment kinetics without feedback such that the membrane-bound fraction of Cdc24 will distribute uniformly.

Let us conclude the model description with several additional remarks:

- A second (Bem1-independent) positive feedback loop for the GEF (Cdc24) has been hypothesized in previous literature [33, 34]. We do not incorporate such a feedback loop in our model. Moreover, we show in Sec. 5.1 that our model can provide an explanation for the experimental findings of [33] without the hypothesized second GEF feedback loop. If they are present, such feedback loops would impart additional redundancy on the Cdc42 polarization machinery and thus add to the robustness of this machinery against genetic perturbations. Identifying these feedback loops in experiments and accounting for them in mathematical models would be an interesting future extension of our work.

- We use the protein dosages for Cdc42, Bem1, Cdc24 and the GAPs reported in [35] to ensure that the values have been obtained by the same method (termed "in-StageTip") and thus are consistent relative to one another. This consistency is important because the relative ratios of the protein dosages are what matters for operation of the various Cdc42 polarization mechanisms. In contrast, the earlier study [12], used values from a range of several distinct, older sources.

Note that there are some significant differences: The dosages of Cdc42 and Bem1 reported in [35] are larger by factors 3 and 6 compared to the values used in [12].

- As the earlier model from [12], our model includes direct recruitment and activation of Cdc42 from the cytosol to the membrane. Other models (e.g. [36]) don't assume this and instead only incorporate activation of Cdc42 on the membrane by Bem1-GEF complexes. Both types of models are qualitatively identical as both capture the key effect that Bem1-GEF generates a sink for Cdc42-GDP and thus leads to directed (cytosolic) diffusion of Cdc42-GDP towards the polar zone.

- For simplicity, we disregard slow intrinsic nucleotide exchange and intrinsic hydrolysis of Cdc42 (which both were reported on the order of $10^{-3}\,\mathrm{s}^{-1}$, see [13, 37]). As these processes are slow and do not impart nonlinear feedback, including these processes does not change our results qualitatively.

- The model does not incorporate the interaction of the Cdc42 polarization machinery with upstream cues (landmark proteins, pheromone signals) that are important for timing of polarization, bud-site selection (see e.g. [25, 38–40]), and shmoo formation [41]. For conceptual cell-polarity models with two components, it was shown in a recent publication [42] that the ability to exhibit spontaneous polarization is a necessary requirement for the maintenance of stationary patterns that are induced by a spatial cue. We therefore expect that our results may also be relevant for the ability of the Cdc42 polarization machinery to exhibit cue-guided polarization.

2 Linear stability analysis in spherical geometry

We determine the regimes where the protein distribution in the cell polarizes spontaneously using linear stability analysis of the bulk-surface coupled reaction–diffusion equations in spherical geometry (see Fig. S1) as given in the Methods section of the main text.

2.1 Laterally homogeneous steady states

The starting point of linear stability analysis are steady states that have homogeneous membrane concentrations $\nabla_{\mathcal{S}}\widetilde{\mathbf{m}} = 0$, and where the bulk concentrations at the membrane are homogeneous $\nabla_{\mathcal{S}}\widetilde{\mathbf{c}}|_{r=R} = 0$. Because the bulk dynamics are purely diffusive, this boundary condition implies that the radial bulk profiles of such steady states must also be homogeneous, that is, $\widetilde{\mathbf{c}}$ is constant in the entire bulk. The homogeneous steady states are thus determined by the set of equations

$$\boldsymbol{f}(\widetilde{\mathbf{m}}, \widetilde{\mathbf{c}}) = 0, \tag{3}$$

$$\mathbf{g}(\widetilde{\mathbf{m}}, \widetilde{\mathbf{c}}) = 0, \tag{4}$$

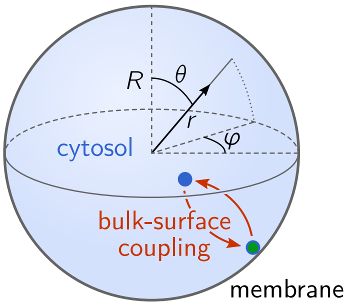

Figure S1. Spherical cell geometry with spherical coordinates (r, φ, θ) and an illustration of the bulk-surface coupling due to attachment–detachment dynamics at the membrane.

together with the total density constraints

$$N_{\mathrm{Cdc42}} = 4\pi R^2(\widetilde{c}_{\mathrm{D}}\, R/3 + \widetilde{m}_{\mathrm{d}} + \widetilde{m}_{\mathrm{t}} + \widetilde{m}_{\mathrm{tg}}), \tag{5a}$$

$$N_{\mathrm{GAPs}} = 4\pi R^2(\widetilde{m}_{\mathrm{g}} + \widetilde{m}_{\mathrm{tg}}), \tag{5b}$$

$$N_{\mathrm{Bem1}} = 4\pi R^2(\widetilde{c}_{\mathrm{B}}\, R/3 + \widetilde{m}_{\mathrm{tb}} + \widetilde{m}_{\mathrm{tbf}}), \tag{5c}$$

$$N_{\mathrm{GEF}} = 4\pi R^2(\widetilde{c}_{\mathrm{F}}\, R/3 + \widetilde{m}_{\mathrm{f}} + \widetilde{m}_{\mathrm{tbf}}). \tag{5d}$$

We solve this set of algebraic equations numerically (implementation in Mathematica using the built-in function `NSolve[]`).

2.2 Linearized dynamics

Linear stability of a steady state is studied by calculating the growth rate of small perturbations $(\delta\mathbf{m}, \delta\mathbf{c})$ around the steady state. For sufficiently small perturbations, the dynamics can be linearized

$$\partial_t \delta\mathbf{c}(r, \varphi, \theta, t) = \mathbf{D}_c \nabla^2 \delta\mathbf{c}, \tag{6}$$

$$-\mathbf{D}_c \partial_r \delta\mathbf{c}|_{r=R} = \boldsymbol{f}_{\mathbf{m}} \delta\mathbf{m} + \boldsymbol{f}_{\mathbf{c}} \delta\mathbf{c}|_{r=R}, \tag{7}$$

$$\partial_t \delta\mathbf{m}(\varphi, \theta, t) = D_m \nabla_{\mathcal{S}}^2|_{r=R} \delta\mathbf{m} + \mathbf{g}_{\mathbf{m}} \delta\mathbf{m} + \mathbf{g}_{\mathbf{c}} \delta\mathbf{c}|_{r=R}, \tag{8}$$

where the matrices $\boldsymbol{f}_{\mathbf{c},\mathbf{m}}$ and $\mathbf{g}_{\mathbf{c},\mathbf{m}}$ are the linearized attachment-detachment kinetics and membrane reactions,

$$\boldsymbol{f}_{\mathbf{c},\mathbf{m}} = \partial_{\mathbf{c},\mathbf{m}} \boldsymbol{f}\big|_{(\widetilde{\mathbf{c}},\widetilde{\mathbf{m}})}, \quad \mathbf{g}_{\mathbf{c},\mathbf{m}} = \partial_{\mathbf{c},\mathbf{m}} \mathbf{g}\big|_{(\widetilde{\mathbf{c}},\widetilde{\mathbf{m}})}, \tag{9}$$

evaluated at the steady state $(\widetilde{\mathbf{c}}, \widetilde{\mathbf{m}})$. In the case of a homogeneous steady state, these matrix coefficients are constant in space. This allows us to find the spatial eigenmodes of the linearized dynamics analytically and reduce the set of linear PDEs (6)–(8) to an algebraic problem that then can be solved numerically.

2.3 Spatial eigenmodes and finding their growth rates

The key idea to solve the linearized dynamics Eqs. (6)–(8) is to consider *elementary perturbations* of the form [12, 43]

$$\delta\mathbf{c}(r,\theta,\varphi,t) = \Phi_c(r,\theta,\varphi)\, e^{\sigma t}\, \delta\hat{\mathbf{c}}, \tag{10a}$$

$$\delta\mathbf{m}(\theta,\varphi,t) = \Phi_m(\theta,\varphi)\, e^{\sigma t}\, \delta\widehat{\mathbf{m}}, \tag{10b}$$

where $\Phi_{c,r}$ are the *spatial eigenmodes* that encode the spatial form of the perturbation and σ is its temporal growth rate. The goal is to find triplets (σ, Φ_c, Φ_m) that fulfill Eqs. (6)–(8). In principle, a general solution of the linearized dynamics can be constructed from a superposition of elementary perturbations. However, we are mostly interested in the stability of the steady state which is determined by the growth rate with the largest real part. If it is positive, the steady state is *linearly unstable* because the corresponding elementary perturbation will grow exponentially in time.

To solve the linear PDEs (6)–(8), the ansatz Eq. (10) requires that the spatial eigenmodes $\Phi_m(\theta,\varphi)$, $\Phi_c(\theta,\varphi,r)$ simultaneously diagonalizes all spatial derivative operators encoding (i) diffusion in the bulk, (ii) diffusion on the membrane, and (iii) the bulk-boundary coupling. Because the spherical geometry obeys the rotational symmetries of the diffusion operators in the bulk and on the surface, we can find such spatial eigenmodes by a separation of variables

$$\Phi_c(\theta,\varphi,r) = \rho(r)\, \Phi_m(\theta,\varphi). \tag{11}$$

Inserting this ansatz into the diffusive bulk dynamics yields[1]

$$\sigma\rho(r)\Phi_m(\theta,\varphi) = D_c\rho(r)\nabla_{\mathcal{S}}^2\Phi_m(\theta,\varphi) + D_c\, r^{-2}\partial_r[r^2\partial_r\rho(r)]\Phi_m(\theta,\varphi).$$

Because there are no mixed derivatives, we can rewrite the previous equation as

$$r^2\sigma - D_c\frac{\partial_r\big(r^2\rho'(r)\big)}{\rho(r)} = r^2\frac{\nabla_{\mathcal{S}}^2\Phi_m(\theta,\varphi)}{\Phi_m(\theta,\varphi)} = -l(l+1) = \text{const}\,, \tag{12}$$

where we introduced the separation constant $-l(l+1)$ such that the angular eigenmodes are the spherical harmonics Y_l^q that solve

$$r^2\nabla_{\mathcal{S}}^2 Y_l^q(\theta,\varphi) = -l(l+1)Y_l^q(\theta,\varphi),$$

where $l \in \mathbb{N}$ and $q \in [-l,l] \subset \mathbb{Z}$ are the 'degree' and 'order' of the spherical harmonic [44]. Essentially, the degree determines the number of peaks in the function Y_l^q, whereas the order q determines where these peaks are located on the sphere. Due to the system's rotational symmetry, only the degree l enters in the linear stability problem. The mode $l = 0$ is uniform, modes with $l = 1$ correspond to a polar patterns, modes with $l = 2$ correspond to bi-polar patterns and so on.

[1] To keep the notation compact, we present the detailed derivation for the case of identical cytosolic diffusion constants. Generalization to different cytosolic diffusion constants follows the same steps where each cytosolic component has its own bulk profile $\rho_i(r)$.

For the radial bulk profile $\rho(r)$, Eq. (12) mandates

$$0 = \partial_r\left[r^2\partial_r\rho(r)\right] - \left[(\sigma/D_c)r^2 + l(l+1)\right]\rho(r),$$

which, using the rescaled radius $\hat{r} = \sqrt{\sigma/D_c}\, r$, is identical to the modified spherical Bessel equation, which are solved by the modified spherical Bessel functions of the first kind $i_l(r)$ [44]. (The spherical Bessel functions of the second kind diverge at $r = 0$ and are therefore unphysical in the setting considered here.) With the convenient normalization $\rho(R) = 1$, the radial bulk profiles are given by

$$\rho(r) = i_l\left(\sqrt{\sigma/D_c}\, r\right) \Big/ i_l\left(\sqrt{\sigma/D_c}\, R\right).$$

Substituting this bulk solution with the ansatz Eq. (10) into the linearized bulk-surface coupling Eq. (7) and membrane dynamics Eq. (8) yields

$$\underbrace{\begin{pmatrix} -\mathbf{D}_c\,\Gamma(\sigma,l) + \boldsymbol{f}_{\mathbf{c}} & \boldsymbol{f}_{\mathbf{m}} \\ \mathbf{g}_{\mathbf{c}} & -\sigma - D_m\frac{l(l+1)}{R^2} + \mathbf{g}_{\mathbf{m}} \end{pmatrix}}_{=:\,\mathbf{M}(\sigma,l)} \begin{pmatrix} \delta\hat{\mathbf{c}} \\ \delta\widehat{\mathbf{m}} \end{pmatrix} = 0, \tag{13}$$

with the bulk-surface coupling coefficient

$$\Gamma(\sigma,l) := \sqrt{\sigma/D_c}\,\frac{i_l'\left(\sqrt{\sigma/D_c}\, R\right)}{i_l\left(\sqrt{\sigma/D_c}\, R\right)}.$$

Using the relationship $i_l(x) = \sqrt{\pi/(2x)}\, I_{l+1/2}(x)$, where $I_k(x)$ denotes the modified Bessel functions of the first kind, one can further evaluate Γ to

$$\Gamma(\sigma,l) = \frac{l}{R} + \sqrt{\sigma/D_c}\,\frac{I_{l+3/2}\left(\sqrt{\sigma/D_c}\, R\right)}{I_{l+1/2}\left(\sqrt{\sigma/D_c}\, R\right)}. \tag{14}$$

In the general case of different cytosolic diffusion constants, $D_{c,i}$ (index i denoting cytosolic components), the bulk-surface coupling coefficient is a diagonal matrix $\mathbf{\Gamma} = \mathrm{diag}(\{\Gamma_i\})$ where D_c is replaced the respective component's bulk diffusivity $D_{c,i}$ in each Γ_i.

The system of linear equations Eq. (13) has non-trivial solutions $(\delta\hat{\mathbf{c}}, \delta\widehat{\mathbf{m}})$ only when the determinant of the matrix $\mathbf{M}(\sigma,l)$ vanishes. Hence, the growth rates σ_l of spatial perturbations with the degree l are determined by the complex solutions of the solvability condition (characteristic equation)

$$\det \mathbf{M}(\sigma_l, l) = 0. \tag{15}$$

The stability of a spatial perturbation is determined by the respective growth rate σ_l with the largest real part. We solve this problem numerically using the iterative

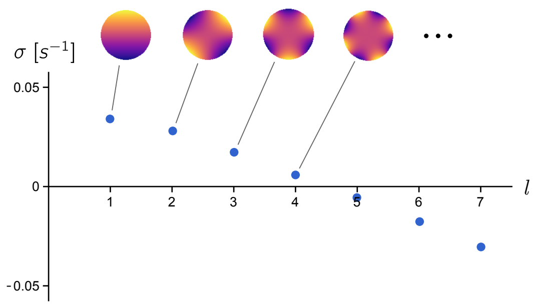

Figure S2. Example for a dispersion relation in spherical geometry, showing the growth rate σ as a function of the spherical harmonic order l. The density plots above illustrate the spherical harmonics with $l = 1...4$.

Newton method (implementation in Mathematica; code provided in the repository `https://github.com/f-brauns/yeast-polarity-LSA`). An example of the resulting relation between the spherical harmonic order l and the dominant growth rate σ_l is shown in Fig. S2. We have used this linear stability analysis to identify the parameter regimes that exhibit spontaneous Cdc42 polarization, i.e. where the homogeneous steady state is laterally unstable (see Sec. 4 below and the stability diagrams in Figs. 2, 4 and 5 in the main text).

3 Numerical simulations

To validate our findings from linear stability analysis and determine the eventual steady state patterns formed, we performed numerical simulations in COMSOL Multiphysics 5.4 (setup files provided in `https://github.com/f-brauns/yeast-polarity-LSA`). This software uses Finite Element Methods (FEM) and has out-of-the-box support for systems with bulk-surface coupling. Simulation results for the various mutations and conditions that were studied experimentally are shown in Movies 1–6. The simulations are initialized with Cdc42, GEF and Bem1 distributed uniformly in the cytosol, with a small random perturbation added (5% random noise). The GAP density is initialized uniformly on the membrane. The parameters used for the numerical simulations are given in Tables S2 and S4, where the former table contains all parameters that have been directly determined in experiments and the latter table states the kinetic rates that were determined by parameter sampling as described in Sec. 4.

In addition, we performed a numerical simulation that emulates the optogenetic recruitment of Cdc24 to a spot on the membrane in *bem1*Δ cells [33]. The optogenetic recruitment is implemented via a spatially dependent increase of Cdc24-attachment rate to $1.0\,\mu\mathrm{m\,s}^{-1}$ in the shape of a Gaussian pulse with a radius of $1\,\mu\mathrm{m}$ starting at 500 s and ending at 1000 s in the simulation. In agreement with the experiments, we find that this transient, localized GEF recruitment stimulates Cdc42-polarization and

that this polarization is maintained after the localized GEF recruitment is switched off (see Movie 6).

In simulations without the optogenetic cue, the position of the polar zone is random. For visualization of the simulations in Movies 1–6, the camera position was chosen after the simulation had finished in such a way that the polar zone appears in approximately the same position in each video. The different camera positions are apparent from the axis-orientation indicator in the bottom right corner.

4 Inference of reaction rate parameters

In the main text, we reasoned why the the rescue mechanism generically is operational only when the GAP to Cdc42 dosage ratio is below a threshold. The quantitative value of the threshold depends on the parameters in the model, many of which are not directly constrained by experiments. However, because we know from previous experiments that polarization is impaired in *bem1*Δ but operational in *bem1*Δ *bem3*Δ [45], we can put bounds on the critical GAP-Cdc42 ratio from above and below: Based on the protein dosages reported in [35], cf. Table S2, the critical ratio lies above $(N_{\mathrm{GAPs}} - N_{\mathrm{Bem3}})/N_{\mathrm{Cdc42}} \approx 0.18$ and below $N_{\mathrm{GAPs}}/N_{\mathrm{Cdc42}} \approx 0.25$. Based on this estimate directly from previous experiments, we can make predictions on the effect of changing the Cdc42 dosage (via a GAL promoter) on different mutant strains, without the need to specify parameter values for the model.

We still need to show that the experimentally found critical GAP-Cdc42 ratio is actually exhibited by the mathematical model for *physiologically realistic* parameter values. To that end, we fix those parameters that are directly constrained by experimental measurements, and perform massive random sampling of the remaining parameters, filtering for those parameter sets that are consistent with our experimental findings on Cdc42 dosage dependence of cell polarity of WT, *bem1*Δ and *bem1*Δ*bem3*Δ cells, as well as the previous experimental observation that a permanently membrane-bound Cdc42 mutant (Cdc42-rit^{C}) is able to polarize [23, 25]. The technical details of this parameter sampling procedure are provided in *Sampling procedure* the subsequent paragraphs below. The Mathemtica code implementing this procedure is available in the supplementary file `parameter-filtering.nb` in the repository `https://github.com/f-brauns/yeast-polarity-LSA`.

Importantly, we find that almost all parameter can be varied over the entire sampled range (four orders of magnitude) indicating that the model is sloppy [46, 47]. For such models, the collective behavior can usually not be used to infer (tightly constrain) the underlying parameters [48]. From the parameter sets identified by the large scale sampling, we picked one representative example to generate the stability diagrams shown in the main text (Figs. 2 and 5). In addition, we filtered under the extra condition of permanently membrane-bound, immobilized Cdc42 to demonstrate that a polarization mechanism based on Bem1–GEF redistribution and local cycling of Cdc42 between active and inactive states (Fig. 4H) can coexist in overlapping parameter regimes with the other two polarization mechanisms (WT and rescue). A representative example out of the parameter sets obtained with this additional condition was used to generate

Name	Unit	Description
k_{D}	$\mu\mathrm{m\,s^{-1}}$	Attachment of cytosolic Cdc42-GDP to the membrane.
k_{tD}	$\mu\mathrm{m^3\,s^{-1}}$	Effective membrane-recruitment of cytosolic Cdc42-GDP by membrane-bound Cdc42-GTP.
k_{d}	$\mathrm{s^{-1}}$	Detachment of inactive Cdc42 from the membrane.
k_{tg}	$\mu\mathrm{m^2\,s^{-1}}$	Cdc42–GAP complex formation on the membrane.
k_{gt}	$\mathrm{s^{-1}}$	Hydrolysis of Cdc42 in the Cdc42–GAP complex and subsequent dissociation of the complex into membrane-bound free GAP and Cdc42-GDP.
k_{tB}	$\mu\mathrm{m^3\,s^{-1}}$	Recruitment of Bem1 by membrane-bound Cdc42-GTP.
k_{b}	$\mathrm{s^{-1}}$	Detachment of Bem1 from the membrane.
k_{bF}	$\mu\mathrm{m^3\,s^{-1}}$	Bem1–GEF complex formation on the membrane by recruitment of GEF to Bem1.
k_{bf}	$\mathrm{s^{-1}}$	Dissociation of Bem1–GEF complexes, releasing GEF into the cytosol.
k_{bfd}	$\mu\mathrm{m^2\,s^{-1}}$	Cdc42 nucleotide exchange driven by membrane-bound Bem1–GEF complexes.
k_{bfD}	$\mu\mathrm{m^3\,s^{-1}}$	Recruitment of Cdc42 from the cytosol by Bem1–GEF complexes.
k_{F}	$\mu\mathrm{m\,s^{-1}}$	Attachment of GEF from the cytosol.
k_{f}	$\mathrm{s^{-1}}$	Detachment of (free) membrane-bound GEF.
k_{fd}	$\mu\mathrm{m^2\,s^{-1}}$	Cdc42 nucleotide exchange driven by (free) membrane-bound GEF.

Table S1. Reaction rates with descriptions. The parameter values used to exemplify the results and a detailed discussion how these values were chosen are provided in Sec. 4.

the stability diagrams shown in Fig. 5C–E.

Sampling procedure. Each of the 13 reaction rates (see Tab. S1) is sampled over four orders of magnitude from 10^{-3} to 10^{1} uniformly on a logarithmic scale. To allow for exact reproduction of the random sampling, the random number generator in Mathematica is seeded with a specified number. For each parameter set, the homogeneous steady state and its dispersion relation (linear stability) are computed under various conditions that emulate the mutants that were studied experimentally (see Tab. S3). The growth rate of the first mode serves as an indicator whether the parameter set exhibits spontaneous polarization under the respective conditions. This is then compared to the experimental observation (cells polarize or not). To provide some intuition how much the different experimental findings in different conditions constrain the parameters, we perform the filtering in subsequent steps for groups of conditions (separated by horizontal lines in Table S3).

Step I: "Core" conditions. We start by filtering for parameter sets consistent with our experiments on WT, *bem1*Δ, and *bem1*Δ *bem3*Δ cells with galactose-induced Cdc42 (conditions 1–6 in Table S3). Out of 5×10^{6} randomly generated parameter sets, 7730 fulfill conditions 1–6, corresponding to a fraction of 0.15%. While this may seem like a small fraction, one should keep in mind that the sampled parameter space is 13-dimensional. If the parameters were all equally important and independent, each of them could be varied across 60% of its full range (four orders of magnitude), since $0.15\% \approx 0.6^{13}$. This illustrates the "curse of dimensionality." Of course in the real system, some of the parameters are not independent, and some of them are more important (stronger constrained) than others.

Step II: Growth of a single Cdc42-cluster in WT. Under WT conditions, the first mode (first spherical harmonic) should grow fastest, ensuring formation of a single Cdc42-cluster ("singularity"). Of the 7730 parameter sets obtained in Step I, 1422 fulfill this condition. Note that there might be specific mechanisms to ensure "singularity" in the real system that are not captured by our model. Candidates for this are vesicle-based transport [50], which we modeled in an effective, coarse-grained way (see Sec. 1.1), and negative feedback via the phosphorylation of Cdc24 by Cla4 [36].

Step III: Permanently membrane-bound Cdc42. Experiments using a permanently membrane-bound Cdc42 mutant (Cdc42-rit^{C}) showed that recycling of Cdc42 via the cytosol is not required for Cdc42 polarization [23, 25]. We wondered whether our mathematical model could reproduce this experimental finding. The permanent membrane-binding of the Cdc42-rit^{C} mutants is accounted for by setting the Cdc42-detachment rate k_{d} to zero (see constraint 8 in Table S3). Out of the previously filtered 1422 parameter sets, 358 fulfill this additional condition. In passing, we note that setting k_{d} to a small but non-zero value does not change this result. In fact, any small k_{d} will benefit cell polarization by allowing a fraction of Cdc42 to diffuse rapidly in the cytosol which accelerates accumulation in the polar zone.

Parameter	Value	Description / Comments
R	$3.5\,\mu m$	Cell radius. For the parameter sampling, we checked that larger cell size, $R = 7\,\mu m$, alone does *not* rescue polarization of *bem1*Δ cells.
N_{Cdc42}	8690	Cdc42 dosage (WT) [35].
N_{Bem1}	1037	Bem1 dosage (WT) [35].
N_{GEF}	934	Cdc24 dosage (WT) [35].
N_{GAPs}	2170	Aggregate dosage of all four Cdc42-GAPs. The individual numbers are 117, 332, 1122, and 599 for Rga1, Rga2, Bem2, and Bem3, respectively [35].
D_c	$11\,\mu m^2\,s^{-1}$	Cytosolic diffusion constants [49].
D_{m_d}	$0.2\,\mu m^2\,s^{-1}$	Diffusion of membrane-bound Cdc42-GDP [23].
$D_{m_\mathrm{t}}, D_{m_\mathrm{tg}}, D_{m_\mathrm{g}}, D_{m_\mathrm{b}}, D_{m_\mathrm{bf}}, D_{m_\mathrm{f}}$	$0.01\,\mu m^2\,s^{-1}$	Diffusion of the remaining membrane-bound proteins. There is no consensus on the membrane diffusion rates in the literature. We use a value that lies in-between the two values $0.03\,\mu m^2\,s^{-1}$, and $0.0025\,\mu m^2\,s^{-1}$ used in previous models [11, 12, 36].
k_{bfd}	$2 \cdot k_{\mathrm{fd}}$	The GEF activity of Bem1–Cdc24 complexes increased approx. two-fold compared to the GEF activity of Cdc24 alone [32].

Table S2. Parameters based on literature values (directly determined by experiments). Note that the protein dosages are stated for wild-type (W)T cells. To account for genetic perturbations (mutations), the values are adapted as specified in Table S3.

#	Polarizes	Experimental mutant / condition	Modified parameters
**1**	yes	WT	—
**2**	no	*bem1*Δ	$N_{\mathrm{Bem1}} = 0$
**3**	no	*bem1*Δ, cell grown to radius 7 µm	$N_{\mathrm{Bem1}} = 0, R = 7\,\mu\mathrm{m}$
**4**	yes	*bem1*Δ *bem3*Δ	$N_{\mathrm{Bem1}} = 0$,<br>$N_{\mathrm{GAPs}} = N_{\mathrm{GAPs}}^{\mathrm{WT}} - N_{\mathrm{Bem3}}$
**5**	yes	*bem1*Δ *GAL–Cdc42*,<br>in 0.1 % Gal (≈ 1.5× Cdc42)	$N_{\mathrm{Bem1}} = 0$,<br>$N_{\mathrm{Cdc42}} = 1.5 \times N_{\mathrm{Cdc42}}^{\mathrm{WT}}$
**6**	yes	*GAL–CDC42* after<br>24 h in 0 % Gal (ca. 20% Cdc42 remaining)	$N_{\mathrm{Cdc42}} = 0.2 \times N_{\mathrm{Cdc42}}^{\mathrm{WT}}$
7	yes	WT (Filtering under the condition that the first mode grows fastest which ensures formation of a single Cdc42-cluster [12], sometimes referred to as "singularity" [2].)	—
8	yes	Cdc42-rit^{C}	$k_{\mathrm{d}} = 0$
9	(yes)	Cdc42-psy1^{TM} (This mutant has been found to polarize in the fission yeast *S. pombe*, but was not yet studied in budding yeast; see text for details)	$k_{\mathrm{d}} = 0$,<br>$D_{m_{\mathrm{d}}} = D_{m_{\mathrm{t}}} = D_{m_{\mathrm{tg}}} = D_{m_{\mathrm{b}}} = D_{m_{\mathrm{bf}}} = 0.0025\,\mu\mathrm{m}^2\,\mathrm{s}^{-1}$

Table S3. Conditions for the filtering of randomly sampled parameters based on experimental findings from this study (1–6, highlighted in bold) and the previous works [23, 25] for Cdc42-rit^{C} (8) and Cdc42-psy1^{TM} in *S. pombe* (9). The criterion for polarization is that the growth rate of the first spherical harmonic is larger than $10^{-4}\,\mathrm{s}^{-1}$, i.e. if $\mathrm{Re}\,\sigma_1 > 10^{-4}\,\mathrm{s}^{-1}$. A parameter set is classified as not polarizing if the growth rates of the first mode is negative (which implies that all higher harmonics also decay because the instability is always a long wavelength instability). Note that since we do not distinguish between different GAPs in the model, the *BEM3* knockout amounts to a reduction of the total GAP dosage by the Bem3 dosage: $N_{\mathrm{GAPs}}^{\mathrm{WT}} - N_{\mathrm{Bem3}} = 2170 - 599 = 1571$. Condition 3 ensures that cell growth alone does not rescue spontaneous polarization of *bem1*Δ cells (the protein dosages are scaled up proportionally to the cell volume, thus keeping the concentrations constant).

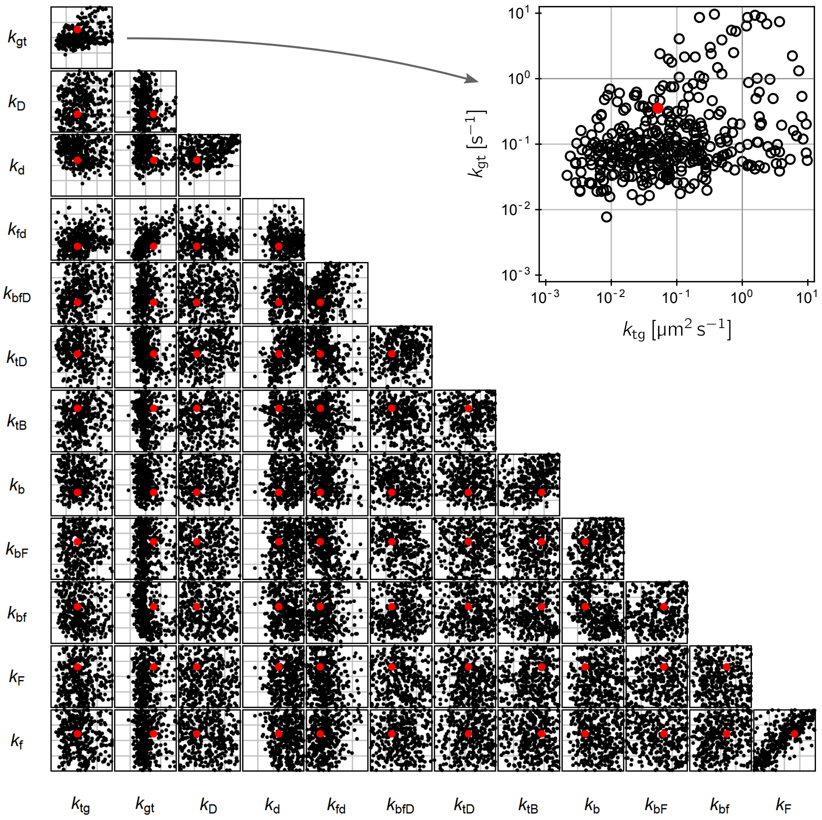

Figure S3. Scatter plots for all pairwise combinations of reaction rates showing the parameter sets that fulfill conditions 1–8 in Table S3 obtained by filtering 5×10^6 randomly generated parameter sets. This parameter set is used in Figs. 2 and 5 and in the Movies 1–6. The red dot marks the parameter set that is closest to the mean of these parameter sets. The inset in the top right corner shows a magnification of the (k_{tg}, k_{gt})-parameter plane. Notably, even the parameter that is constrained the strongest (k_{gt}, the dissociation rate of Cdc42-GAP complexes) ranges over several order of magnitude.

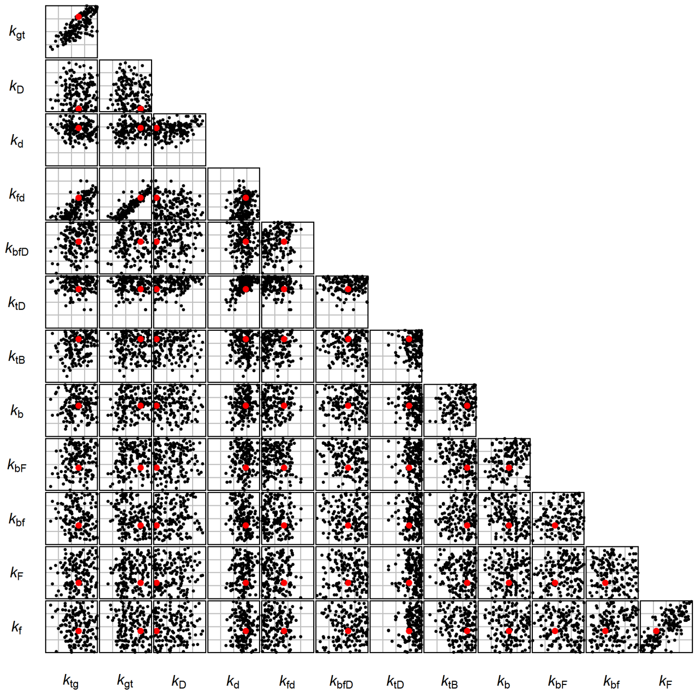

Figure S4. Pairwise scatter plots of the parameter sets that fulfill conditions 1–6 and 9 in Table S3 (out of 5×10^6 randomly generated parameter sets). The red dot marks the parameter set that is closest to the mean. This parameter set is used in Fig. 4.

To visualize the filtered parameter sets, we show scatter plots for each pair of parameters (see Fig. S3). This shows how much each parameter is constrained by the experiments and visually highlights pair-wise correlations in the parameter sets.

In summary, we find that parameter sets consistent with the experimental observations cover a large range of parameters. Most parameters can vary over the full four orders of magnitude we sampled. Even the most strongly confined parameters, k_d (Cdc42-GDP membrane dissociation rate) and k_gt (hydrolysis rate of Cdc42 in Cdc42-GAP complexes) each cover more than two orders of magnitude.

Polarization independent of spatial Cdc42 redistribution. The analysis of the Cdc42-polarization machinery in terms of functional subunits carried out in the main text revealed that there might be a polarization mechanism that is independent of spatial Cdc42 redistribution (Fig. 4H). Instead, polarization relies on redistribution of Bem1-GEF complexes and GAP saturation to the polar zone. Cdc42 merely switches between its active and inactive state, while its *total density* remains spatially uniform. To test where this mechanism is operational, we introduce a new condition (9

Name	Figs. 2 and 5	Fig. 4	Unit
k_{D}	0.015	0.0017	$\mu m\, s^{-1}$
k_{tD}	0.17	1.0	$\mu m^3\, s^{-1}$
k_{d}	0.22	0.82	s^{-1}
k_{tg}	0.053	0.36	$\mu m^2\, s^{-1}$
k_{gt}	0.35	1.5	s^{-1}
k_{tB}	0.68	2.1	$\mu m^3\, s^{-1}$
k_{b}	0.032	0.23	s^{-1}
k_{bF}	0.29	0.057	$\mu m^3\, s^{-1}$
k_{bf}	0.25	0.03	s^{-1}
k_{bfD}	0.025	0.3	$\mu m^3\, s^{-1}$
k_{F}	0.43	0.017	$\mu m\, s^{-1}$
k_{f}	0.26	0.046	s^{-1}
k_{fd}	0.0077	0.052	$\mu m^2\, s^{-1}$

Table S4. Parameters (kinetic rates described in Tab. S1) obtained by filtering for parameter sets that reproduce the experimental findings. The parameter set used for Figs. 2 and 5 is marked by a red dot in Fig. S3. It was obtained by filtering with conditions 1–8 (Table S3) that correspond to experiments performed with budding yeast and this study and [23]. The parameter set used for Fig. 4, marked by a red dot in Fig. S4, was generated separately to demonstrate that *theoretically* all three mechanisms of polarization encoded in the Cdc42-interaction network can coexist in the same parameter regime. To this end, filtering was performed with conditions 1–6 and 9. The experiment with the membrane-bound, immobile Cdc42 mutant Cdc42-psy1^{TM}, corresponding to condition 9, was so far only performed in fission yeast, not in budding yeast, justifying the use of a different parameter set that is viable in this condition too.

in Table S3). Under this condition, all Cdc42 is membrane bound (no detachment, $k_\mathrm{d} = 0$) and transport of Cdc42 on the membrane is suppressed by setting the diffusion constants of both active and inactive membrane-bound Cdc42 to the same small value ($0.0025\,\mu\mathrm{m}^2\,\mathrm{s}^{-1}$, based on an estimate from experiments [51]). Since for equal diffusion constants the dynamics of Cdc42's *total density* decouples and is purely diffusive, it remains uniform at all times.

Interestingly, out of the 358 parameter sets obtained in Step III above (fulfilling conditions 1–8 in Table S3 only 33 exhibit spontaneous polarization with immobile Cdc42 (condition 9). This suggests that it is unlikely that Cdc42-psy1$^\mathrm{TM}$ mutants are able to polarize in budding yeast. However, polarization of Cdc42-psy1$^\mathrm{TM}$ has been found in fission yeast [23], which exhibits a bi-polar polarization pattern in WT cells. We therefore sampled parameters using only conditions 1–6 and 9, i.e. without the constraint that a single polar cap emerges under WT conditions. The 155 parameter sets found this way are shown in Fig. S4. This demonstrates that the Cdc42-transport independent mechanism could, in principle, be operational in the same parameter regime as the WT and rescue mechanisms. For the phase diagrams shown in Fig. 4 in the main text, we used the parameter set that is closest to the mean of all parameter sets fulfilling conditions 1–6 and 9. A simulation showing pattern formation for the Cdc42-psy1$^\mathrm{TM}$ mutant conditions is provided in the file `params-2_Cdc42-psy1TM.mph`.

5 Supplementary Discussion

5.1 Previous experiments explained by our model

Permanently membrane-bound Cdc42 mutants. Our model reproduces experiments with Cdc42-rit$^\mathrm{C}$ fusion chimeras that are permanently membrane-bound [23], as we have shown in Sec. 4. The only means of Cdc42 transport in these mutants is owing to the slower diffusion of active Cdc42 compared to inactive Cdc42 on the membrane. The formation of complexes between Cdc42-GTP and Gic1/Gic2 has been suggested as a molecular mechanism underlying the slow diffusion of Cdc42-GTP [24, 25]. Indeed, Kang et al. find synthetic lethality of *gic1*Δ *gic2*Δ, Cdc42-rit$^\mathrm{C}$ triple mutants and conclude: "*Therefore, polarization of Cdc42-ritC is likely to be mediated by Gics during the first phase of G1.* [...] *A possible explanation for the synthetic lethality of cdc42-rit^C gic1Δ gic2Δ is that even a slight increase of its mobility (in the absence of Gic1/2) could be more detrimental to polarization of Cdc42-rit^C, which presumably occurs via lateral diffusion and/or GDI-independent exchanges between membrane and cytosol.*" Our model and theoretical analysis suggest that Cdc42-ritC polarization requires differential diffusion of Cdc42-GTP vs Cdc42-GDP on the membrane to achieve redistribution of total Cdc42. The Gics provide this differential diffusion as they effectively reduce the diffusivity of Cdc42-GTP compared to Cdc42-GDP. This corroborates the above explanation by Kang et al. why one of the Gics is required for polarization of cdc42-rit$^\mathrm{C}$ mutants.

Another strongly membrane-bound Cdc42 mutant, fused to a trans-membrane domain (Cdc42-psy1$^\mathrm{TM}$), was studied in *S. pombe* (fission yeast) [52]. Whereas Cdc42-

rit^C binds to the membrane by an amphipathic helix and therefore can diffuse rather freely on the membrane surface, the transmembrane domain of Cdc42-psy1^{TM} renders this fusion chimera effectively immobile. Strikingly, it was found that Cdc42-psy1^{TM} exhibits polarization of Cdc42 activity without Cdc42 accumulation [23]. Since the Cdc42-polarization machinery shares the key components with budding yeast (see [53] for a review), we hypothesize that this puzzling observation could be explained by our mathematical model of the Cdc42-polarization machinery. In particular, Scd1 and Scd2 (paralogs of Bem1 and Cdc24) facilitate the same scaffold-mediated feedback loop that operates in WT budding yeast. Our mathematical analysis shows that the scaffold-mediated feedback loop in conjunction with GAP-saturation can facilitate polarization independently of Cdc42-redistribution (see Fig. 4H in the main text). Moreover, our parameter study in Sec. 4 above shows that this mechanism can, in principle, be operational in the same regime as the WT mechanism. In contrast to the WT mechanism, our model predicts that polarization with immobile Cdc42 is sensitive to the GAP-Cdc42 dosage ratio because it relies on GAP-saturation in the polar zone; see Fig. 4E,H in the main text.

Optogenetic GEF recruitment. In a recent set of experiments in budding yeast, optogenetics was used to transiently recruit Cdc24 to a small membrane patch [33]. Interestingly it was found that in *bem1*Δ cells, this optogenetic GEF-localization can induce stable patterns that are maintained even after the optogenetic "stimulus" is removed. This suggests that the *bem1*Δ cells are in a *subcritical* regime, where a sufficiently strong local perturbation (stimulus) can induce self-sustained polarization. Previous theoretical work has shown that subcriticality is generic in mass-conserving reaction–diffusion systems and that the regimes of stimulus-induced pattern formation are always adjacent to regimes of spontaneous pattern formation. [42, 54]. We therefore expect that the rescue mechanism supports stimulus-induced pattern formation above the critical GAP:Cdc42 threshold in *bem1*Δ cells. Indeed, and in agreement with the experimental findings reported in [33], numerical simulations of *bem1*Δ mutants emulating the transient optogenetic recruitment of GEF to a small membrane patch, show that polarization is maintained after the stimulus removed (see Movie 5). In conclusion, the rescue mechanism can explain the experimentally found, stimulus-induced polarization of Cdc42, independently of a feedback loop operating on the GEF.

Globally enhanced GEF activity rescues Bem1-deletes. Previous experiments have shown that Bem1-mutants that lack the Cdc42-GTP interaction domain but still interact with Cdc24 rescue polarization of *bem1*Δ cells [28, 55]. It has been hypothesized that this is because these Bem1-mutants relieve Cdc24's autoinhibition and thereby globally increase GEF activity. In accordance with this, the Bem1-independent rescue mechanism predicted by our model can be activated by a global increase of GEF activity (see Fig. 2B in the main text).

In another set of experiments, Bem1's ability to localize Cdc24 to the zone of high Cdc42-GTP concentration was inhibited by fusing Bem1 to the strongly membrane

bound Snc2 [10]. Strikingly, this Bem1-Snc2^{V39A,M42A} fusion chimera does not rescue cell polarization. As a reason for this, we hypothesize that the interaction between Bem1-Snc2^{V39A,M42A} and Cdc42 sequesters part of the Cdc42 in the cell and lowers the available amount of Cdc42 below the threshold where the Bem1-independent mechanism is operational. In agreement with this hypothesis, recent results show that sequestration of a fraction of Cdc42 to the membrane by overexpression of the Ccd42-binding protein Gic2 is lethal for *bem1*Δ *bem3*Δ cells [28].

5.2 Hypothetical feedback via Rsr1 and secondary GEFs

Several proteins from the bud-site selection pathway have been hypothesized to mediate positive feedback loops that may give rise to spontaneous Cdc42-polarization. A hypothetical interaction of the Rsr1-GEF Bud5 with Cdc42 has been suggested recently [34] based on the observation that Bud5 relocalizes from the landmark-determined ring that surrounds the bud-scar to clusters of Cdc42-GTP [56, 57]. This may mediate a feedback loop between Cdc42 and Rsr1 via their respective GEFs Cdc24 and Bud5, that facilitates spontaneous polarization similarly to the Bem1–Cdc42 mutual recruitment mechanism. A similar hypothetical feedback loop mediated by the Rsr1-GEF Bud3 has been suggested [8].

Such feedback loops would further contribute to the redundancy of polarization mechanisms in budding yeast because Rsr1 could effectively take on the role of Bem1. However, because proteins suggested to mediate these feedback loops bind to (axial) landmark proteins that localize to the bud scar, it is likely that the feedback would be strongly localized in the vicinity of the bud scar. It was previously observed that Cdc42 polarization in *bem1*Δ*bem3*Δ cells is not preferentially directed to the vicinity of the bud scar [45]. This indicates that landmark associated proteins, such as Rsr1, Bud3, and Bud5 do not play a central role in the rescue mechanism. That these proteins do not play an essential role in the rescue of *bem1*Δ cells is further supported by the finding that *bem1*Δ*rsr1*Δ are viable in some strain backgrounds [55]. Further experiments will be required in the future to elucidate the interplay between the bud-site selection pathway and spontaneous polarization of Cdc42.

6 Experiments

6.1 Growth frequency assay (Fig. 3)

In the growth assay shown in Fig. 3 growth rates were determined for various genotypes/galactose concentration combinations. However, not all combinations exhibit the same frequency of growth. Table S5 shows how often growth occurred within the total number of replicates performed.

Error bars in Fig. 3 show the 68% confidence interval for the mean. Large error bars result from two effects: (i) For *Gal1-sfGFP-Cdc42 BEM1 BEM3*, they originate from the technical replicate variation, i.e., noise across runs/wells. (ii) For the *bem1*Δ at low galactose concentrations, there is very infrequent growth. For instance, at 0.03%

	WT*			GFP*			M1*			M2*		
% Gal	TG	#R	#E	TG	#R	#E	TG	#R	#E	TG	#R	#E
0	10	10	4	0	4	2	0	6	2	0	4	2
0.01	10	10	4	4	4	2	0	6	2	0	4	2
0.015	10	10	4	4	4	2	0	6	2	0	4	2
0.02	10	10	4	4	4	2	0	6	2	0	4	2
0.03	10	10	4	4	4	2	1	6	2	0	4	2
0.04	10	10	4	4	4	2	0	6	2	0	4	2
0.05	10	10	4	4	4	2	0	6	2	0	4	2
0.06	10	10	4	4	4	2	1	6	2	1	4	2
0.08	10	10	4	4	4	2	0	6	2	1	4	2
0.10	10	10	4	4	4	2	4	6	2	1	4	2
0.20	9	10	4	4	4	2	6	6	2	4	4	2
2.00	8	8	3	2	2	1	6	6	2	2	2	1

Table S5. Number of wells where growth is observed for various genotypes and conditions of the data underlying Fig. 3. TG indicates the number of runs (#R) that grew across #E experiments.* Strain abbreviations: WT: *CDC42*, GFP: p_{GAL1}-*CDC42-sfGFPSW*, M1: *bem1*Δ p_{GAL1}-*CDC42-sfGFP*^{SW}, M2: *bem1*Δ *bem3*Δ p_{GAL1}-*CDC42-sfGFP*^{SW}.

there was growth only in one well. In this case, the variance of only one Student t likelihood, which depends on the fitting error corrected for overdispersion, is the main determinant for the width of the credible interval, so this makes this interval a bit wide. However, the poor fitness of this background will likely still yield large credible intervals even with a large number of replicates, as its low fitness also makes it vulnerable for suppressor sweeps of the population. The stochasticity in growth at low galactose concentrations for *bem1*Δ backgrounds is also seen in the other data points (see Table S6).

6.2 Determination of fitness effects of sfGFP-CDC42 sandwich fusion

Additional growth rate assays were performed to determine possible growth rate defects of sfGFP tagging of Cdc42. These were taken in a Biotek Epoch™ 2 Microstrain Spectrophotometer strain reader, a different type compared to that of the assay of Fig. 3. All strain backgrounds contained *BEM1* and *BEM3*. This assay compares the relative performance of a p_{GAL1}-*Cdc42-sfGFP*^{SW} strain compared to the p_{GAL1}-*CDC42* strain within this experiment. From Fig. S5, we can see that growth with and without sfGFP occurs at similar rates. The growth rate deteriorates notably when reducing induction for the strains with galactose-dependent Cdc42 production. However, we also witnessed that when fitness is low, there is a realistic risk that late in the measurements a relatively fast-growing population emerges in our wells.

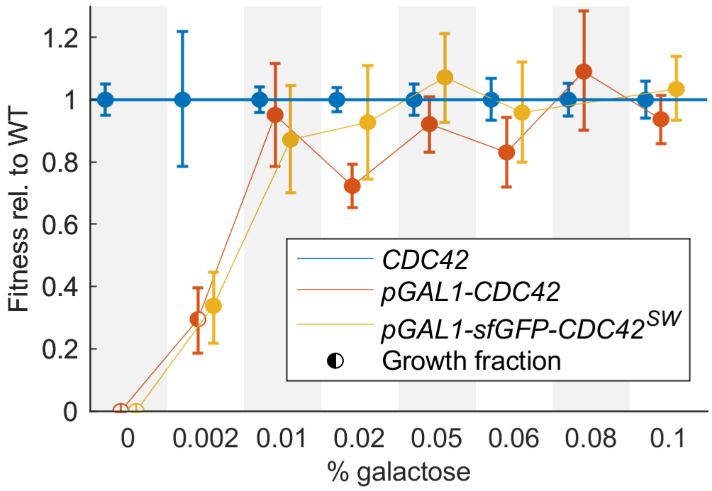

Figure S5. Normalized fitness data for different CDC42 variants (indicated by colors) with suppressor filtering. All backgrounds are with *BEM1* and *BEM3*. Markers indicate the mean of the posterior probability distribution for the fitness and error bars mark the 95% credible interval. Pie chart marker shows how frequently growth occurs across replicates (filled circle corresponding to 100%). The number of experiments per strain/condition pair is given in Supplementary Table S7.

	CDC42				p_{GAL1}-*CDC42*				p_{GAL1}-*CDC42*-*sfGFP*^{SW}			
% Gal	#D	TG	#R	#E	#D	TG	#R	#E	#D	TG	#R	#E
0	4	8	8	6	12	0	18	9	9	0	3	4
0.002	0	2	2	1	0	1	2	1	0	1	1	1
0.01	2	10	10	6	10	2	2	6	0	2	2	2
0.02	1	11	11	6	6	6	6	6	0	2	2	2
0.05	1	9	9	5	3	7	7	5	0	1	1	1
0.06	0	8	8	4	3	7	7	5	1	3	3	3
0.08	0	4	4	2	3	1	1	2	–	–	–	–
0.10	2	10	10	6	3	19	19	9	0	8	8	4

Table S6. Number of wells where growth is observed for the *CDC42* variants in the growth assay data with suppressor filtering. All backgrounds are with *BEM1* and *BEM3*. The column #D denotes how many wells with growth have been marked as potentially from suppressors and are thus discarded from the analysis, the remainder of the growing wells are in the columns named times (TG) growth and runs (#R). #E denotes the number of experiments that comprise all runs (#R) including those discarded ones.

We suspect that mutants carrying a suppressor, presumably concerning the galactose induction system, arise which sweep the population. Therefore, we apply a coarse filter in an at-tempt to remove these, at least partially. For this purpose, we fit all optical density (OD) curves with a sigmoidal curve, which allows us to infer to initial OD. As during incubation colonies are growing to saturation to an OD of around 1, we expect an initial OD of about 0.01. Consequently, those wells that suggest an inferred OD at time = 0 that is more than an order of magnitude lower (< 0.001), are discarded. We confirm that we are not too relaxed with our threshold as on occasion a WT well is discarded which is unlikely to have been swept by a mutant. Indeed, we see the galactose-dependent strains have higher rates of suspected suppressor growth (see Table S6) and we see the bulk of the suppressors are detected when fitness is low (see also Fig. S5). It is important to note that while this particularly cleans the growth rate data for low galactose concentrations, this filtering is usually inconsequential for the question whether sfGFP addition influences fitness (Table S6). Using as our metric the Bayes factor (posterior odds ratio) that the strain which is observed to grow faster is actually faster against the opposite statement, we require a value > 10 for strong evidence [58]. After filtering, there is never strong evidence for significant growth rate difference between having sfGFP or not.

6.3 Size and viability assays under variable expression of CDC42

To further substantiate that the differences in population growth rates are directly caused by the ability of cells to polarize, rather than for example pleiotropic changes in another cell cycle phase, we measured the cell radius using light microscopy (Fig. S7A).

% Gal	0.002	0.01	0.02	0.05	0.06	0.1
Bayes factor without filtering	2	22	7	4	7	4
Bayes factor with filtering	2	2	7	5	3	5

Table S7. Posterior odds ratio (Bayes factor) per medium condition of the probability that the strain with observed faster growth (between the p_{GAL1}-*CDC42* and p_{GAL1}-*CDC42-sfGFP*^{SW} strains) is actually faster, divided by the probability that the opposite is true. We distinguish the cases where we aim to filter suppressor growth away from our data sets and the case where we do not.

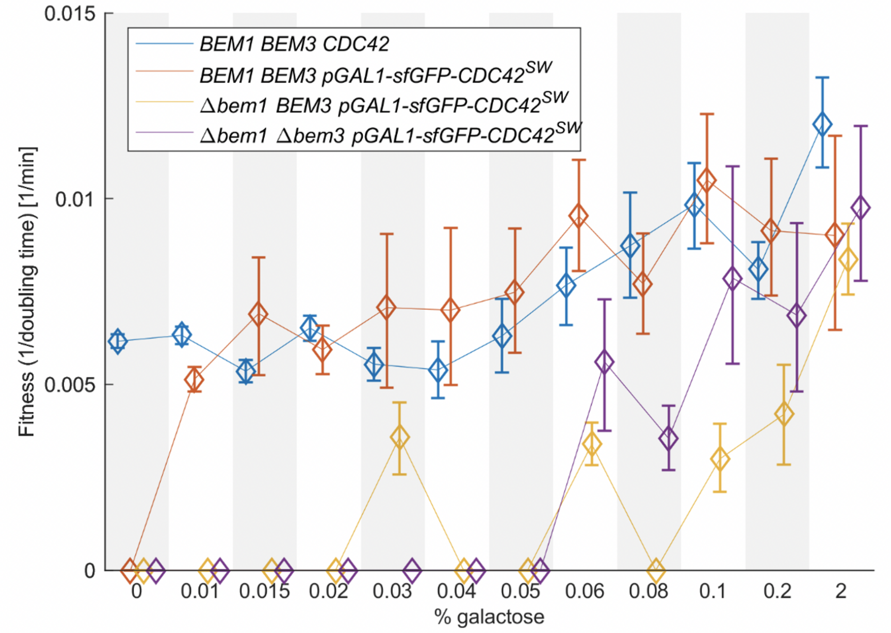

Figure S6. Non-normalized growth rates of the different mutant strains against galactose concentration (relates to Fig. 3 in the main text). Markers show the mean of the posterior probability distribution for the fitness and the error bars indicate the 68% credible interval; see Methods section in the main text. The number of experiments per strain/condition pair is given in Supplementary Table S6.

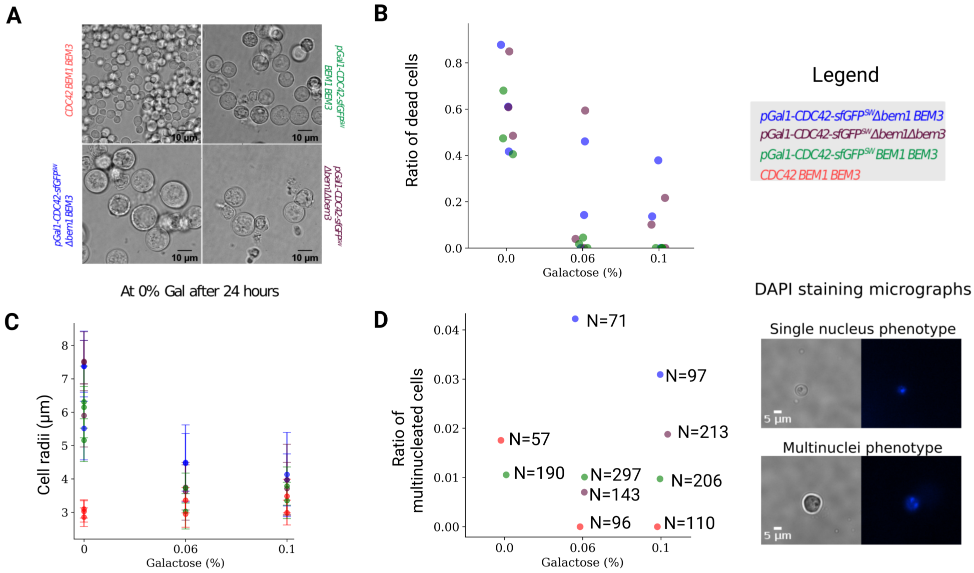

Figure S7. Morphology and viability for WT and mutant strains with pGAL1-CDC42-sfGFPSW at various induction levels. **A** Microscopy images show the morphology of dead and alive cells after 24 h at 0% galactose concentration, resulting in a Cdc42 dosage that approximates zero; scale bar indicates 10 µm. **B** Ratio of dead cells of the relevant mutants over different galactose concentrations. The data contains information from three independent experiments. **C** Cell radii of the relevant mutants over different galactose concentrations. Each point represents the arithmetic mean of the cell radii per technical replicate (every independent experiment). Error bars represent the standard deviation of each dataset. **D** Ratio of multinucleated cells of the relevant mutants over different galactose concentrations. The data contains information from one experiment per genotype.

All microscopy was performed with a Nikon Eclipse Ti-E inverted microscope with an oil immersion 60X objective with NA 1.40 and refractive index of 1.51. The software used for data collection was Nis-Elements Advanced research version 4.51. Data collection of brightfield microscopy images were acquire over three different galactose concentrations (0% , 0.06% and 0.1%) after 24h of incubation of the strains: ywkd065, ywkd069, ywkd070 and yll3a.

It was previously shown that the cell radius correlates linearly with the time it takes for cells to polarize [45, 59]: cells that take longer to polarize are on average larger than cells that polarize fast because yeast cells continue to grow during polarity establishment, allowing us to use the cell radius as a proxy for the polarization time. Additionally, we verified that, at low Cdc42 dosages, cells cannot polarize at all and thus die. Consistent with the population growth data, we observed that after 24 hours at 0% galactose concentration, for every genetic background where Cdc42 is under the galactose promotor, the vast majority of cells are not able to polarize or polarize very slowly, because they are either dead (Fig. S7A–C) or very large (Fig. S7A, D). We also confirm that the average cell radius (and thus the polarization time) and death rate of cells with Cdc42 under its native promotor are not affected by the galactose concentration (Fig. S7C, D in red). At 0.06% galactose concentration, *bem1*Δ *bem3*Δ cells' radii (and thus polarization times) are closer to WT cell radii than those of *bem1*Δ cells. This agrees with the population growth data. And at 0.1% galactose concentration, the average cell radius for live cells for all mutants were approximately equal to the average WT cell radius (Fig. S7D). Interestingly, after 24 hours at 0% galactose concentration, WT cells with Cdc42 under the galactose promotor are still polarizing faster than the *bem1*Δ and the *bem1*Δ*bem3*Δ cells, as indicated by their smaller average cell radius (Fig. S7D). This observation confirms our above observation that a very small number of Cdc42 molecules is sufficient for WT cells to polarize and thus for the WT mechanism to be operational.

6.4 Multinucleated and dead cells

Cells that do not polarize are predicted to have nuclear divisions but no cellular division and therefore, we hypothesize that cells with polarization defects are more likely to be multinucleated.

Multinucleated cells. We measured the percentage of multinucleated cells for the different genetic backgrounds and galactose concentrations using DAPI staining. The DAPI staining protocol was taken from [60]. Two experiments failed due to two causes: too little staining for the amount of analysed cells and DAPI stain saturation for taking a long time (> 30 min) from the staining to the microscope. In the last situation most of the cells were stained and not only the nuclei. Samples were imaged in a cover slip right after the DAPI staining. The emission wavelength was 450 nm and the excitation wavelength was 395 nm. The exposure time was 70ms and the laser power was 1%. Data analysis was performed with the Cell Counter plugin from ImageJ (v1.53t). Each cell was labelled to count the total number of cells in each frame. Further, each cell with two or more nuclei and the dead cells were differently labelled. After DAPI

staining we do not observe any cells at 0% galactose concentration for the *bem1*Δ and *bem1*Δ*bem3*Δ cells that were alive before staining (dead cells are permeable and overstain for DAPI), and thus we also do not observe any multinucleated cells. These results also suggest that we underestimate the number of dead cells when we use bright field microscopy as in Fig. S7. The wild-type backgrounds with and without the galactose promoter show a low percentage of multinucleated cells. As the galactose concentration goes up the fraction of dead cells for the *bem1*Δ and *bem1*Δ*bem3*Δ mutants goes down. The number of detected multinucleated cells remains low. We interpret that our results are consistent with the hypothesis that multinucleated cells die more rapidly in stationary phase than single nucleated cells and therefor are hard to detect in this assay.

Cell radii. We used the "oval tool" to encircle every cell and measure its perimeter, using the "Analyse" → "Measure" tool. From the perimeters p, the cell radii were computed using the formula $r = p/(2\pi)$.

Dead cells. To identify dead cells, we looked at the following morphological features [61]: cell shrinkage, swelling of organelles, nuclear fragmentation and plasma membrane blebbing.

Supplementary References

[1] Wu, H., Rossi, G. & Brennwald, P. The ghost in the machine: Small GTPases as spatial regulators of exocytosis. *Trends in Cell Biology* **18**, 397–404 (2008). URL https://linkinghub.elsevier.com/retrieve/pii/S096289240800189X.

[2] Howell, A. S. *et al.* Singularity in Polarization: Rewiring Yeast Cells to Make Two Buds. *Cell* **139**, 731–743 (2009). URL https://www.cell.com/cell/abstract/S0092-8674(09)01314-2.

[3] Layton, A. T. *et al.* Modeling Vesicle Traffic Reveals Unexpected Consequences for Cdc42p-Mediated Polarity Establishment. *Current Biology* **21**, 184–194 (2011). URL https://linkinghub.elsevier.com/retrieve/pii/S0960982211000352.

[4] Watson, L. J., Rossi, G. & Brennwald, P. Quantitative Analysis of Membrane Trafficking in Regulation of Cdc42 Polarity. *Traffic* **15**, 1330–1343 (2014). URL http://doi.wiley.com/10.1111/tra.12211.

[5] Woods, B. *et al.* Parallel Actin-Independent Recycling Pathways Polarize Cdc42 in Budding Yeast. *Current Biology* **26**, 2114–2126 (2016). URL https://linkinghub.elsevier.com/retrieve/pii/S0960982216306881.

[6] Fairn, G. D., Hermansson, M., Somerharju, P. & Grinstein, S. Phosphatidylserine is polarized and required for proper Cdc42 localization and for development of cell polarity. *Nature Cell Biology* **13**, 1424–1430 (2011). URL http://www.nature.com/articles/ncb2351.

[7] Okada, S. *et al.* Daughter Cell Identity Emerges from the Interplay of Cdc42, Septins, and Exocytosis. *Developmental Cell* **26**, 148–161 (2013). URL https://linkinghub.elsevier.com/retrieve/pii/S1534580713003547.

[8] Martin, S. G. Spontaneous cell polarization: Feedback control of Cdc42 GTPase breaks cellular symmetry. *BioEssays* **37**, 1193–1201 (2015). URL http://doi.wiley.com/10.1002/bies.201500077.

[9] Chen, H. *et al.* Cdc42p regulation of the yeast formin Bni1p mediated by the effector Gic2p. *Molecular Biology of the Cell* **23**, 3814–3826 (2012). URL https://www.molbiolcell.org/doi/10.1091/mbc.e12-05-0400.

[10] Woods, B., Kuo, C.-C., Wu, C.-F., Zyla, T. R. & Lew, D. J. Polarity establishment requires localized activation of Cdc42. *The Journal of Cell Biology* **211**, 19–26 (2015). URL http://jcb.rupress.org/content/211/1/19.

[11] Goryachev, A. B. & Pokhilko, A. V. Dynamics of Cdc42 network embodies a Turing-type mechanism of yeast cell polarity. *FEBS Letters* **582**, 1437–1443 (2008). URL https://febs.onlinelibrary.wiley.com/doi/abs/10.1016/j.febslet.2008.03.029.

[12] Klünder, B., Freisinger, T., Wedlich-Söldner, R. & Frey, E. GDI-Mediated Cell Polarization in Yeast Provides Precise Spatial and Temporal Control of Cdc42 Signaling. *PLOS Computational Biology* **9**, e1003396 (2013). URL https://journals.plos.org/ploscompbiol/article?id=10.1371/journal.pcbi.1003396.

[13] Zhang, B., Wang, Z.-X. & Zheng, Y. Characterization of the Interactions between the Small GTPase Cdc42 and Its GTPase-activating Proteins and Putative Effectors. *Journal of Biological Chemistry* **272**, 21999–22007 (1997). URL http://www.jbc.org/lookup/doi/10.1074/jbc.272.35.21999.

[14] Khalili, B., Merlini, L., Vincenzetti, V., Martin, S. G. & Vavylonis, D. Exploration and stabilization of Ras1 mating zone: A mechanism with positive and negative feedbacks. *PLOS Computational Biology* **14**, e1006317 (2018). URL https://dx.plos.org/10.1371/journal.pcbi.1006317.

[15] Howell, A. S. *et al.* Negative Feedback Enhances Robustness in the Yeast Polarity Establishment Circuit. *Cell* **149**, 322–333 (2012). URL https://linkinghub.elsevier.com/retrieve/pii/S009286741200342X.

[16] Bi, E. & Park, H.-O. Cell Polarization and Cytokinesis in Budding Yeast. *Genetics* **191**, 347–387 (2012). URL http://www.genetics.org/lookup/doi/10.1534/genetics.111.132886.

[17] Stevenson, B. J. *et al.* Mutation of RGA1, which encodes a putative GTPase-activating protein for the polarity-establishment protein Cdc42p, activates the pheromone-response pathway in the yeast Saccharomyces cerevisiae. *Genes &*

Development **9**, 2949–2963 (1995). URL http://www.genesdev.org/cgi/doi/10.1101/gad.9.23.2949.

[18] Mukherjee, D. *et al.* Bem3, a Cdc42 GTPase-activating protein, traffics to an intracellular compartment and recruits the secretory Rab GTPase Sec4 to endomembranes. *Journal of Cell Science* **126**, 4560–4571 (2013). URL http://jcs.biologists.org/cgi/doi/10.1242/jcs.117663.

[19] Tong, Z. *et al.* Adjacent positioning of cellular structures enabled by a Cdc42 GTPase-activating protein–mediated zone of inhibition. *The Journal of Cell Biology* **179**, 1375–1384 (2007). URL http://jcb.rupress.org/content/179/7/1375.

[20] Knaus, M. *et al.* Phosphorylation of Bem2p and Bem3p may contribute to local activation of Cdc42p at bud emergence. *The EMBO Journal* **26**, 4501–4513 (2007). URL https://www.embopress.org/doi/abs/10.1038/sj.emboj.7601873.

[21] Caviston, J. P., Longtine, M., Pringle, J. R. & Bi, E. The Role of Cdc42p GTPase-activating Proteins in Assembly of the Septin Ring in Yeast. *Molecular Biology of the Cell* **14**, 4051–4066 (2003). URL https://www.molbiolcell.org/doi/10.1091/mbc.e03-04-0247.

[22] Tiedje, C., Sakwa, I., Just, U. & Höfken, T. The Rho GDI Rdi1 Regulates Rho GTPases by Distinct Mechanisms. *Molecular Biology of the Cell* **19**, 2885–2896 (2008). URL https://www.molbiolcell.org/doi/10.1091/mbc.e07-11-1152.

[23] Bendezú, F. O. *et al.* Spontaneous Cdc42 Polarization Independent of GDI-Mediated Extraction and Actin-Based Trafficking. *PLOS Biology* **13**, e1002097 (2015). URL https://journals.plos.org/plosbiology/article?id=10.1371/journal.pbio.1002097.

[24] Daniels, C. N., Zyla, T. R. & Lew, D. J. A role for Gic1 and Gic2 in Cdc42 polarization at elevated temperature. *PLOS ONE* **13**, e0200863 (2018). URL http://dx.plos.org/10.1371/journal.pone.0200863.

[25] Kang, P. J., Miller, K. E., Gueguenlat, J., Beven, L. & Park, H.-O. The shared role of the Rsr1 GTPase and Gic1/Gic2 in Cdc42 polarization. *Molecular Biology of the Cell* **29**, 2359–2369 (2018). URL https://www.molbiolcell.org/doi/10.1091/mbc.E18-02-0145.

[26] Das, A. *et al.* Flippase-mediated phospholipid asymmetry promotes fast Cdc42 recycling in dynamic maintenance of cell polarity. *Nature Cell Biology* **14**, 304–310 (2012). URL http://www.nature.com/articles/ncb2444.

[27] Brown, J. L., Jaquenoud, M., Gulli, M.-P., Chant, J. & Peter, M. Novel Cdc42-binding proteins Gic1 and Gic2 control cell polarity in yeast. *Genes & Development* **11**, 2972–2982 (1997). URL http://www.genesdev.org/cgi/doi/10.1101/gad.11.22.2972.

[28] Grinhagens, S. *et al.* A time-resolved interaction analysis of Bem1 reconstructs the flow of Cdc42 during polar growth. *Life Science Alliance* **3**, e202000813 (2020). URL https://www.life-science-alliance.org/lookup/doi/10.26508/lsa.202000813.

[29] Chen, G.-C., Kim, Y.-J. & Chan, C. S. The Cdc42 GTPase-associated proteins Gic1 and Gic2 are required for polarized cell growth in Saccharomyces cerevisiae. *Genes & Development* **11**, 2958–2971 (1997). URL http://www.genesdev.org/cgi/doi/10.1101/gad.11.22.2958.

[30] Rossman, K. L., Der, C. J. & Sondek, J. GEF means go: Turning on RHO GTPases with guanine nucleotide-exchange factors. *Nature Reviews Molecular Cell Biology* **6**, 167–180 (2005). URL http://www.nature.com/articles/nrm1587.

[31] Rapali, P. *et al.* Scaffold-mediated gating of Cdc42 signalling flux. *eLife* **6**, e25257 (2017). URL https://elifesciences.org/articles/25257.

[32] Shimada, Y., Wiget, P., Gulli, M.-P., Bi, E. & Peter, M. The nucleotide exchange factor Cdc24p may be regulated by auto-inhibition. *The EMBO Journal* **23**, 1051–1062 (2004). URL http://emboj.embopress.org/cgi/doi/10.1038/sj.emboj.7600124.

[33] Witte, K., Strickland, D. & Glotzer, M. Cell cycle entry triggers a switch between two modes of Cdc42 activation during yeast polarization. *eLife* **6**, e26722 (2017). URL https://doi.org/10.7554/eLife.26722.

[34] Goryachev, A. B. & Leda, M. Many roads to symmetry breaking: Molecular mechanisms and theoretical models of yeast cell polarity. *Molecular Biology of the Cell* **28**, 370–380 (2017). URL https://www.molbiolcell.org/doi/10.1091/mbc.e16-10-0739.

[35] Kulak, N. A., Pichler, G., Paron, I., Nagaraj, N. & Mann, M. Minimal, encapsulated proteomic-sample processing applied to copy-number estimation in eukaryotic cells. *Nature Methods* **11**, 319–324 (2014). URL http://www.nature.com/articles/nmeth.2834.

[36] Kuo, C.-C. *et al.* Inhibitory GEF Phosphorylation Provides Negative Feedback in the Yeast Polarity Circuit. *Current Biology* **24**, 753–759 (2014). URL https://linkinghub.elsevier.com/retrieve/pii/S0960982214001924.

[37] Zheng, Y., Cerione, R. & Bender, A. Control of the yeast bud-site assembly GTPase Cdc42. Catalysis of guanine nucleotide exchange by Cdc24 and stimulation of GTPase activity by Bem3. *The Journal of Biological Chemistry* **269**, 2369–2372 (1994).

[38] Miller, K. E., Lo, W.-C., Lee, M. E., Kang, P. J. & Park, H.-O. Fine-tuning the orientation of the polarity axis by Rga1, a Cdc42 GTPase-activating protein. *Molecular Biology of the Cell* **28**, 3773–3788 (2017). URL https://www.molbiolcell.org/doi/10.1091/mbc.e17-01-0074.

[39] Kang, P. J., Lee, M. E. & Park, H.-O. Bud3 activates Cdc42 to establish a proper growth site in budding yeast. *The Journal of Cell Biology* **206**, 19–28 (2014). URL http://www.jcb.org/lookup/doi/10.1083/jcb.201402040.

[40] Miller, K. E., Lo, W.-C., Chou, C.-S. & Park, H.-O. Temporal regulation of cell polarity via the interaction of the Ras GTPase Rsr1 and the scaffold protein Bem1. *Molecular Biology of the Cell* **30**, 2543–2557 (2019). URL https://www.molbiolcell.org/doi/10.1091/mbc.E19-02-0106.

[41] Muller, N. *et al.* A Predictive Model for Yeast Cell Polarization in Pheromone Gradients. *PLOS Computational Biology* **12**, e1004795 (2016). URL https://dx.plos.org/10.1371/journal.pcbi.1004795.

[42] Brauns, F., Halatek, J. & Frey, E. Phase-Space Geometry of Mass-Conserving Reaction-Diffusion Dynamics. *Physical Review X* **10**, 041036 (2020). URL https://link.aps.org/doi/10.1103/PhysRevX.10.041036.

[43] Levine, H. & Rappel, W.-J. Membrane-bound Turing patterns. *Physical Review E* **72**, 061912 (2005). URL https://link.aps.org/doi/10.1103/PhysRevE.72.061912.

[44] Bronshtein, I. N. *Handbook of mathematics* (Springer, Berlin; New York, 2007). URL http://www.myilibrary.com?id=135274.

[45] Laan, L., Koschwanez, J. H. & Murray, A. W. Evolutionary adaptation after crippling cell polarization follows reproducible trajectories. *eLife* **4**, e09638 (2015). URL https://elifesciences.org/articles/09638.

[46] Gutenkunst, R. N. *et al.* Universally Sloppy Parameter Sensitivities in Systems Biology Models. *PLoS Computational Biology* **3**, e189 (2007). URL https://dx.plos.org/10.1371/journal.pcbi.0030189.

[47] Daniels, B. C., Chen, Y.-J., Sethna, J. P., Gutenkunst, R. N. & Myers, C. R. Sloppiness, robustness, and evolvability in systems biology. *Current Opinion in Biotechnology* **19**, 389–395 (2008). URL https://linkinghub.elsevier.com/retrieve/pii/S0958166908000785.

[48] Transtrum, M. K., Machta, B. B. & Sethna, J. P. Why are Nonlinear Fits to Data so Challenging? *Physical Review Letters* **104**, 060201 (2010). URL https://link.aps.org/doi/10.1103/PhysRevLett.104.060201.

[49] Slaughter, B. D., Schwartz, J. W. & Li, R. Mapping dynamic protein interactions in MAP kinase signaling using live-cell fluorescence fluctuation spectroscopy and imaging. *Proceedings of the National Academy of Sciences* **104**, 20320–20325 (2007). URL http://www.pnas.org/cgi/doi/10.1073/pnas.0710336105.

[50] Freisinger, T. *et al.* Establishment of a robust single axis of cell polarity by coupling multiple positive feedback loops. *Nature Communications* **4**, 1807 (2013). URL http://www.nature.com/articles/ncomms2795.

[51] Valdez-Taubas, J. & Pelham, H. R. B. Slow Diffusion of Proteins in the Yeast Plasma Membrane Allows Polarity to Be Maintained by Endocytic Cycling. *Current Biology* **13**, 1636–1640 (2003). URL http://www.sciencedirect.com/science/article/pii/S0960982203006584.

[52] Bement, W. M. *et al.* Activator–inhibitor coupling between Rho signalling and actin assembly makes the cell cortex an excitable medium. *Nature Cell Biology* **17**, 1471–1483 (2015). URL http://www.nature.com/articles/ncb3251.

[53] Chiou, J.-g., Balasubramanian, M. K. & Lew, D. J. Cell Polarity in Yeast. *Annual Review of Cell and Developmental Biology* **33**, 77–101 (2017). URL http://www.annualreviews.org/doi/10.1146/annurev-cellbio-100616-060856.

[54] Trong, P. K., Nicola, E. M., Goehring, N. W., Kumar, K. V. & Grill, S. W. Parameter-space topology of models for cell polarity. *New Journal of Physics* **16**, 065009 (2014). URL http://stacks.iop.org/1367-2630/16/i=6/a=065009?key=crossref.9a0354f957a7fd3f82a01b89fc8ef283.

[55] Smith, S. E. *et al.* Independence of symmetry breaking on Bem1-mediated autocatalytic activation of Cdc42. *The Journal of Cell Biology* **202**, 1091–1106 (2013). URL http://jcb.rupress.org/content/202/7/1091.

[56] Kang, P. J. A GDP/GTP Exchange Factor Involved in Linking a Spatial Landmark to Cell Polarity. *Science* **292**, 1376–1378 (2001). URL http://www.sciencemag.org/cgi/doi/10.1126/science.1060360.

[57] Marston, A. L., Chen, T., Yang, M. C., Belhumeur, P. & Chant, J. A localized GTPase exchange factor, Bud5, determines the orientation of division axes in yeast. *Current Biology* **11**, 803–807 (2001). URL https://linkinghub.elsevier.com/retrieve/pii/S0960982201002305.

[58] Kass, R. E. & Raftery, A. E. Bayes Factors. *Journal of the American Statistical Association* **90**, 773–795 (1995). URL http://www.tandfonline.com/doi/abs/10.1080/01621459.1995.10476572.

[59] Allard, C. A. H., Decker, F., Weiner, O. D., Toettcher, J. E. & Graziano, B. R. A size-invariant bud-duration timer enables robustness in yeast cell size control. *PLOS ONE* **13**, e0209301 (2018). URL http://dx.plos.org/10.1371/journal.pone.0209301.

[60] Cone, A. Yeast DAPI Staining v1 (2016). URL https://www.protocols.io/view/Yeast-DAPI-Staining-eigbcbw.

[61] Munoz, A. J., Wanichthanarak, K., Meza, E. & Petranovic, D. Systems biology of yeast cell death. *FEMS Yeast Research* **12**, 249–265 (2012). URL https://academic.oup.com/femsyr/article-lookup/doi/10.1111/j.1567-1364.2011.00781.x.
