# Supplementary material for: Redundancy and the role of protein copy numbers in the cell polarization machinery of budding yeast
Source: Nat Commun. 2023 Oct 16;14:6504. doi: 10.1038/s41467-023-42100-0 (PMC10579396; doi:10.1038/s41467-023-42100-0)
Supplement: Supplementary file 3 — Reporting Summary [file 41467_2023_42100_MOESM3_ESM.pdf]

nature portfolio

Corresponding author(s): Liedewij Laan and Erwin Frey

Last updated by author(s): Sep 13, 2023

Reporting Summary

Nature Portfolio wishes to improve the reproducibility of the work that we publish. This form provides structure for consistency and transparency in reporting. For further information on Nature Portfolio policies, see our Editorial Policies and the Editorial Policy Checklist.

Statistics

For all statistical analyses, confirm that the following items are present in the figure legend, table legend, main text, or Methods section.

n/a	Confirmed	
☐	☒	The exact sample size (*n*) for each experimental group/condition, given as a discrete number and unit of measurement
☐	☒	A statement on whether measurements were taken from distinct samples or whether the same sample was measured repeatedly
☐	☒	The statistical test(s) used AND whether they are one- or two-sided<br>*Only common tests should be described solely by name; describe more complex techniques in the Methods section.*
☒	☐	A description of all covariates tested
☐	☒	A description of any assumptions or corrections, such as tests of normality and adjustment for multiple comparisons
☐	☒	A full description of the statistical parameters including central tendency (e.g. means) or other basic estimates (e.g. regression coefficient) AND variation (e.g. standard deviation) or associated estimates of uncertainty (e.g. confidence intervals)
☒	☐	For null hypothesis testing, the test statistic (e.g. *F*, *t*, *r*) with confidence intervals, effect sizes, degrees of freedom and *P* value noted<br>*Give P values as exact values whenever suitable.*
☒	☐	For Bayesian analysis, information on the choice of priors and Markov chain Monte Carlo settings
☒	☐	For hierarchical and complex designs, identification of the appropriate level for tests and full reporting of outcomes
☒	☐	Estimates of effect sizes (e.g. Cohen's *d*, Pearson's *r*), indicating how they were calculated

Our web collection on statistics for biologists contains articles on many of the points above.

Software and code

Policy information about availability of computer code

Data collection	Nis-Elements Advanced v4.51; ImageJ v1.53t
Data analysis	Matlab and Python; Code link: https://github.com/leilaicruz/Experimental-data-analysis-protein-copy-number-in-polarity

For manuscripts utilizing custom algorithms or software that are central to the research but not yet described in published literature, software must be made available to editors and reviewers. We strongly encourage code deposition in a community repository (e.g. GitHub). See the Nature Portfolio guidelines for submitting code & software for further information.

Data

Policy information about availability of data

All manuscripts must include a data availability statement. This statement should provide the following information, where applicable:

- Accession codes, unique identifiers, or web links for publicly available datasets
- A description of any restrictions on data availability
- For clinical datasets or third party data, please ensure that the statement adheres to our policy

Data links: https://doi.org/10.4121/60bea990-b1a6-40c7-9355-584e061791d5.v1, https://doi.org/10.4121/67e56fe8-6b54-446b-aaac-cd2e245ee066.v1

Research involving human participants, their data, or biological material

Policy information about studies with human participants or human data. See also policy information about sex, gender (identity/presentation), and sexual orientation and race, ethnicity and racism.

Reporting on sex and gender	n/a
Reporting on race, ethnicity, or other socially relevant groupings	n/a
Population characteristics	n/a
Recruitment	n/a
Ethics oversight	n/a

Note that full information on the approval of the study protocol must also be provided in the manuscript.

Field-specific reporting

Please select the one below that is the best fit for your research. If you are not sure, read the appropriate sections before making your selection.

[x] Life sciences [ ] Behavioural & social sciences [ ] Ecological, evolutionary & environmental sciences

For a reference copy of the document with all sections, see nature.com/documents/nr-reporting-summary-flat.pdf

Life sciences study design

All studies must disclose on these points even when the disclosure is negative.

Sample size	We did not calculate a sample size beforehand. Our goal was to have three independent experiments to average out variation between experiments. because of experimental problems such as contamination or evaporation we sometimes ended up with two independent experiments/ conditions. However since we have data over different induction concentrations we can still observe a trend.
Data exclusions	One run for Figure 3 had two contaminated controls for the 2% galactose condition, and this condition for all strains in this run was excluded from further analysis. This criterion was pre-established as contamination checks are the purpose of having negative control wells which were not inoculated with our strains.<br>As explained in detail in the Supplementary Information, we encountered problems with presumptive emergence of suppressor mutations in our strains during the experiments. We showed what the effects are of excluding measurements subject to these mutations, and what these selection criteria were (growth rate not in accordance with expected initial cell density). This exclusion was not pre-established.<br>For the experiments on multi-nucleated cells we excluded two experiments because the DAPI did not stain appropriately, once because they were strained too strongly and once because they were stained too weakly.
Replication	The data set in Figure 3 contained at least 4 measurements per strain-condition combination (technical replicates). The number of replicates for all experiments are presented in the SI.
Randomization	We did not randomize how we placed our cells into the 96 well plate since we extensively worked on our assay to reduce evaporation, contamination etc. to ensure that there are no biases within the 96 plate, before we started data collection.
Blinding	Investigators were not blinded to the allocation of strains to growth medium conditions, as we used the allocation information for distributing the strains to the right conditions in our designed lay-out in the experimental wells.

Reporting for specific materials, systems and methods

We require information from authors about some types of materials, experimental systems and methods used in many studies. Here, indicate whether each material, system or method listed is relevant to your study. If you are not sure if a list item applies to your research, read the appropriate section before selecting a response.

Materials & experimental systems

n/a	Involved in the study
☒	☐ Antibodies
☒	☐ Eukaryotic cell lines
☒	☐ Palaeontology and archaeology
☒	☐ Animals and other organisms
☒	☐ Clinical data
☒	☐ Dual use research of concern
☒	☐ Plants

Methods

n/a	Involved in the study
☒	☐ ChIP-seq
☒	☐ Flow cytometry
☒	☐ MRI-based neuroimaging
