# Supplementary material for: Redundancy and the role of protein copy numbers in the cell polarization machinery of budding yeast
Source: Nat Commun. 2023 Oct 16;14:6504. doi: 10.1038/s41467-023-42100-0 (PMC10579396; doi:10.1038/s41467-023-42100-0)
Supplement: Supplementary file 4 — Description of Additional Supplementary Files [file 41467_2023_42100_MOESM4_ESM.pdf]

Description of Additional Supplementary Files

File name: Supplementary movie 1
Description: Simulation under WT conditions, see (1) in Table S3. The density of membrane bound Cdc42-GTP concentration, m_t, is plotted on the surface of the sphere that mimics the cell (radius 3.5 µm). Simulation file: params-1_WT.mph

File name: Supplementary movie 2
Description: Simulation of a bem1Δ bem3Δ mutant, see (4) in Supp. Table S3. The density of membrane bound Cdc42-GTP concentration, m_t, is plotted on the surface of the sphere that mimics the cell (radius 3.5 µm). Simulation file: params-1_bem1d_bem3d.mph

File name: Supplementary movie 3
Description: Simulation of a bem1Δ mutant with 1.5×Cdc42 overexpression, see (5) in Supp. Table S3. The density of membrane bound Cdc42-GTP concentration, m_t, is plotted on the surface of the sphere that mimics the cell (radius 3.5 µm). Simulation file: params-1_bem1d_1.5xCdc42.mph

File name: Supplementary movie 4
Description: Simulation of a wild-type cell with 0.2×Cdc42 underexpression, see (6) in Supp. Table S3. The density of membrane bound Cdc42-GTP concentration, m_t, is plotted on the surface of the sphere that mimics the cell (radius 3.5 µm). Simulation file: params-1_bem1d_0.2xCdc42.mph

File name: Supplementary movie 5
Description: Simulation of Cdc42-ritC mutant, where Cdc42 cannot detach from the membrane, i.e. is transported by lateral diffusion on the membrane alone, see (6) in Supp. Table S3. The density of membrane bound Cdc42-GTP concentration, m_t, is plotted on the surface of the sphere that mimics the cell (radius 3.5 µm). Simulation file: params-1_Cdc42-ritC.mph

File name: Supplementary movie 6
Description: Simulation of a bem1Δ mutant with optognetic GEF recruitment. Left: Visualization of the transient, spatially localized increase in the GEF membrane-attachment rate k_F emulating optogenetic GEF recruitment. The recruitment is switched on in the interval 500 s < t < 1000 s and has the shape of a Gaussian pulse with radius 1 µm. Right: Density plot of the membrane bound Cdc42-GTP concentration; cell radius 3.5 µm. Simulation file: params-1_bem1d GEF-stimulus.mph
